# Thyroid cancer burden and risk factors in China from 1990-2019: a systematic analysis using the global burden of disease study

**DOI:** 10.3389/fonc.2023.1231636

**Published:** 2023-11-08

**Authors:** Shuai Jin, Li Luo, Xiaodong Xu, Kaide Xia

**Affiliations:** ^1^ School of Biology and Engineering (School of Health and Medicine Modern Industry), Guizhou Medical University, Guiyang, China; ^2^ Department of Clinical Laboratory, The Second People’s Hospital of Guiyang, Guiyang, China; ^3^ Hospital Infection Management Department, Bijie First People’s Hospital, Bijie, China; ^4^ Molecular Diagnostic Laboratory, Guiyang Maternal and Child Health Care Hospital, Guiyang, China

**Keywords:** thyroid cancer, global disease burden, high body-mass index, China, DALYs

## Abstract

**Background:**

Thyroid cancer (TC) is the most common endocrine system malignancy with a rapidly increasing incidence in China. Epidemiological data on TC at the national level are lacking. This study aimed to quantify the TC disease burden in China between 1990 and 2019 and evaluate the current status and trends of the disease burden attributed to a high body mass index (HBMI).

**Methods:**

The 2019 Global Burden of Disease Study dataset was used to explore the TC disease burden. Age-standardized rates of incidence (ASIR), prevalence (ASPR), deaths (ASDR), and disability-adjusted life years (DALYs) were considered and the estimated annual percentage change (EAPC) was calculated as a measure of the average change in age-standardized rates. The trend in TC-related mortality and DALYs attributed to an HBMI, accounting for different age groups and sexes, was examined.

**Results:**

Between 1990 and 2019, the ASDR and DALYs for TC decreased by 0.02/100000 and 1.17/100000, respectively. The ASPR and ASIR increased by 9.88/100000 and 1.04/100000, respectively. The EAPC for ASDR, age-standardized rates of DALYs, ASPR, and ASIR were 0.06 (95% CI: -0.09, 0.21), -0.20 (95% CI: -0.31, -0.10), 3.52 (95% CI: 3.35, 3.68), and 2.73 (95% CI: 2.58, 2.88), respectively. TC-related deaths, DALYs, and their prevalence and incidence in China increased by 118%, 350%, 81%, and 290%, respectively. The disease burden of TC was higher among male than female patients in different age groups, with varying distributions. The disease burden attributed to HBMI gradually increased over the past 30 years according to age-standardized DALYs, particularly in male patients.

**Conclusion:**

The TC burden has increased in China over the past 30 years, and population aging poses a challenge to TC prevention and control. HBMI has become an important factor in the TC disease burden and further research should focus on reducing the disease burden among Chinese male patients with TC.

## Introduction

Thyroid cancer (TC) is the most common endocrine tumor worldwide (1). In 2020, the Global Cancer Epidemiology Database reported that TC accounted for 3.0% of new cancer cases worldwide, contributing to 0.4% of cancer deaths (2). The incidence of TC has continued to increase in countries and regions (3) such as North America (4, 5), South America (6), Europe (7), and Asia (8, 9). TC is one of the most common malignant tumors among Chinese women and an 20.1% increase in the annual increase of TC has been reported between 2003 and 2011 (10).

The standards for high body mass index were established through the use of mixed-effects modeling and data adjustment, taking into account factors such as country, year, age, and sex (11). Typically, a body mass index greater than 25 kg/m2 was considered indicative of a high body mass index for adults aged 20 and above (12, 13). High body-mass index (HBMI) is a common indicator of thyroid cancer (14, 15). HBMI increases the risk of developing differentiated thyroid cancer, particularly in women whose body shape changes from lean to fat at the beginning of menstruation (16). HBMI is a well-established risk factor for TC in China (17).

This study examined the current status and trends over the past 30 years of TC and the disease burden associated with high BMI according to sex and age in China. This comprehensive and multifaceted analysis may be beneficial for healthcare planning, health promotion, and resource allocation.

## Methods

### Data source

The Global Burden of Diseases, Injuries, and Risk Factors Study (GBD) is an international collaborative study developed and maintained by the Institute for Health Metrics and Evaluation (IHME) at the University of Washington that aims to provide rigorous and comparable measures for global health problems. The GBD data methodology and results have been previously described (18, 19). The database contains estimated statistics on 369 diseases and 87 risk factors in 204 countries and regions between 1990 and 2019. The 1990-2019 GBD data on TC in China was extracted for analysis. As the dataset was free and open to researchers and did not contain personally identifiable data, ethical approval from the relevant institutional review board and informed consent of patients included in this study were not required.

### Risk factors

The 2019 GBD study utilized a framework of comparative risk assessment to compare mortality and disability-adjusted life-years (DALYs) associated with 87 different risk factors and combinations of such factors across various global levels, countries, and regions (20). These risk factors are divided into four categories: environmental, occupational, metabolic, and behavioral. Of the 87 risk factors evaluated in this study, a HBMI was recognized as a risk factor for TC (21).

### Disease burden

This study utilized prevalence, incidence, mortality, and DALYs to evaluate the disease burden of TC. The nonfatal disease burden was evaluated using DisMod-MR 2.1, a Bayesian meta-regression tool that assesses the available data on disease incidence, prevalence, and mortality for consistency in epidemiological parameters (22). The GBD estimation process involves the identification of various pertinent data sources for each disease or injury, such as inpatient data, national surveys, published studies, and the CDC cause-of-death reporting system (22). These data provide estimates of incidence and prevalence adjusted for readmissions, non-primary diagnoses, and outpatient usage, and form an important basis for the etiologic model. Mortality rates for TC were generated through the application of the cause-of-death integration model, which provides a systematic way to run different mortality or cause-of-death models with various selections of covariates to identify the set of models that best represent all available input data.

DALYs are defined as the total number of healthy life years lost from morbidity to death, summed by the years of life lost due to premature death (YLL) and years of life lost due to disability (YLD). If N represents the number of deaths and L represents the standard life expectancy (in years) at the age of death, YLL is obtained by summing N × L and YLD is obtained by summing I × DW × L, where I denotes the number of cases of incidence, DW is the disability weight, and L is the average number of years of disability. The disability weight indicates the severity of health loss associated with a single health condition.

### Analysis

Between 1950 and 2019, the GBD database used age-standardized rate (ASR) population estimates to average the world population and eliminate the effect of time series on population numbers. ASR was calculated using the following formula (23): ASR= $\frac{\sum_{i=1}^{A}a_{i}w_{i}}{\sum_{i=1}^{A}w_{i}}$ * 100, 000. Age-standardized rates of incidence (ASIR), prevalence (ASPR), deaths (ASDR), and DALYS for the 1990-2019 period were used to describe the disease burden. The central estimates for each measured variable were determined by calculating the mean values of all draws, whereas the range of the 95% uncertainty intervals (UIs) was determined based on the values at the 2.5th and 97.5th percentiles (24). Estimated annual percentage changes (EAPC) are often used to represent annual changes in time trends and can provide more accurate information than percentage changes; therefore, this study used EAPC to describe changes in the disease burden of TC by sex (25). The relevant calculation principle of EAPC has been described as follows: when EAPC and its 95% confidence interval (CI) are greater than 0, ASR increases; when it is less than 0, ASR decreases; if the 95% CI includes 0, ASR remains stable (23, 26). Biaxial and bubble plots were used to present the data and observe the trends in TC burden according to sex and age between 1990 and 2019.

## Results

### Thyroid cancer burden in 1990 and 2019

**Table 1** shows the number and composition of mortality, DALYs, incidence, and prevalence for TC overall and according to sex between 1990 and 2019. Overall, from 1990 to 2019, there has been an increase in the number of TC-related deaths, disability-adjusted life years (DALYs), prevalence, and incidence in China. The number of TC related deaths was 3319 (95% UI:2862-4133) in 1990 and 7239 (95% UI:6012-8476) in 2019, indicating an almost 118% increase in mortality. The number of prevalence increased from 69239 (95% UI:55771-82950) in 1990 to 310328 (95% UI:255041-382138) in 2019, representing an almost 3.5 fold increase in prevalence. The number of DALYs and incidence increased by 81% and 290%, respectively. The 2019 ASPR for TC 16.17 (95% UI:13.28-19.83) and ASIR 2.05 (95% UI:1.70-2.50) showed a significant upward trend. TC-related ASR of DALYs significantly decreased from 10.87 (95% UI:9.33-13.21) in 1990 to 9.70 (95% UI:8.11-11.27) in 2019. In contrast, there was no significant change in the TC-related ASDR between 1990 and 2019 (EAPC=0.06, 95% CI: -0.09-0.21).

**Table 1:** Thyroid cancer burden cases and rates in China in 1990 and 2019.

Sex	Number (95% UI)		ASR per 100000 (95% UI)		EAPC (95% CI)
	1990	2019	1990	2019	
Both					
Deaths	3319(2862,4133)	7239(6012,8476)	0.42(0.37,0.53)	0.39(0.32,0.45)	0.06(-0.09,0.21)
DALYs	103493(87958,124715)	187319(156236,219112)	10.87(9.33,13.21)	9.7(8.11,11.27)	-0.20(-0.31,-0.10)
Prevalence	69239(55771,82950)	310328(255041,382138)	6.29(5.14,7.53)	16.17(13.28,19.83)	3.52(3.35,3.68)
Incidence	10030(8399,11907)	39079(32279,47658)	1.01(0.86,1.21)	2.05(1.70,2.50)	2.73(2.58,2.88)
Male					
Deaths	1230(1004,1524)	4212(3178,5241)	0.35(0.29,0.43)	0.52(0.4,0.64)	2.20(1.86,2.54)
DALYs	38444(31360,46793)	107110(81449,133260)	8.36(6.89,10.21)	11.64(8.91,14.29)	1.84(1.56,2.13)
Prevalence	13847(11230,17098)	118423(89065,149034)	2.46(2,3.04)	12.22(9.29,15.34)	6.70(6.27,7.13)
Incidence	2505(2038,3101)	16111(12076,20192)	0.55(0.45,0.67)	1.74(1.32,2.16)	4.99(4.59,5.39)
Female					
Deaths	2089(1711,2681)	3027(2395,3759)	0.50(0.41,0.63)	0.3(0.24,0.38)	-1.79(-1.86,-1.71)
DALYs	65049(52359,80806)	80208(64737,99491)	13.48(10.98,16.66)	8.11(6.55,10.07)	-2.00(-2.11,-1.89)
Prevalence	55392(42570,69006)	191904(145570,253044)	10.42(8.08,12.91)	20.21(15.32,26.45)	2.20(2.03,2.36)
Incidence	7525(5907,9307)	22968(17457,30160)	1.5(1.19,1.86)	2.41(1.83,3.17)	1.57(1.45,1.68)

ASR, Age-standardized rate; DALYs, Disability-adjusted life-years; EAPC, Estimated annual percentage change; UIs, Uncertainty intervals.

Between 1990 and 2019, all indicators related to the burden of TC in men increased to varying degrees, with the number of prevalence increasing nearly 7.6-fold while mortality, DALYs, and incidence increased 2.4-, 1.8-, and 5.4-fold, respectively. ASIR, ASPR, ASDR, and age-standardized DALYs showed significant increases; the EAPC and its confidence intervals were all greater than 0. TC-related prevalence and incidence in women also showed significant increases, with EAPCs of 2.20 (95% CI:2.03-2.36) and 1.57 (95% CI:1.45-1.68), respectively. In contrast, mortality and DALYs showed a significant decrease, with EAPCs of -1.79 (95% CI: -1.86 to -1.71) and -2.00 (95% CI: -2.11 to -1.89), respectively.

### Thyroid cancer disease burden by age in 2019

**Figure 1:**
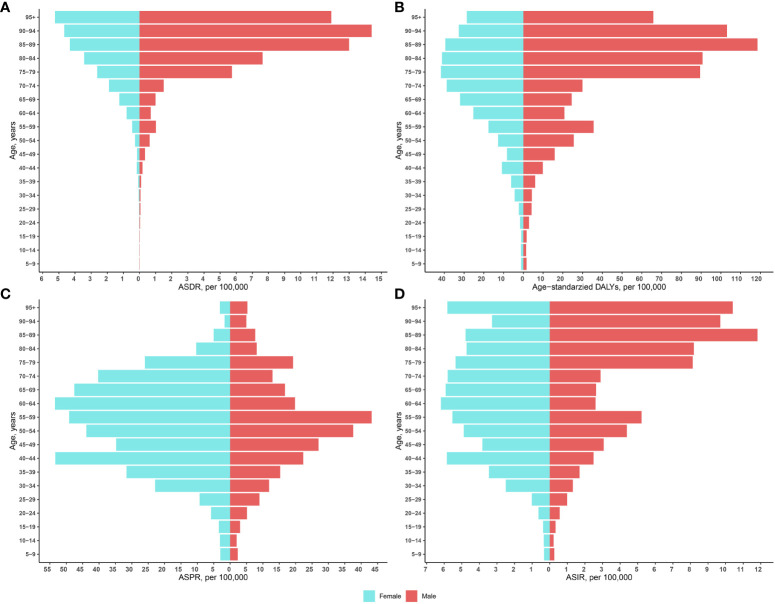
Age-standardized rates of mortality **(A)**, DALYs **(B)**, ASPR **(C)**, and ASIR **(D)** by sex and age in 2019. ASIR, Age-standardized incidence rate; ASDR, Age-standardized deaths rate; ASPR, Age-standardized prevalence rate; DALYs, Disability-adjusted life-years.

**Figure 1** shows the distribution of TC disease burden by age group in 2019. TC rarely causes death in patients aged 0-40 years, and TC-related deaths increase progressively with age in female patients over the age of 40 years. Deaths in male patients were more concentrated above 80 years of age, and the associated deaths were higher than those in female patients (**Figure 1A**). Among age-standardized DALYs, female patients reached a peak at age 75-79 years at 41.7/100000. Male patients showed a distinct bimodal feature, that is, the first peak occurred at age 55-59 years, 35.6/100000, and the second peak was 118.5/100000 at age 85-89 years. Age-corrected DALYs for TC were significantly higher in men than in women (**Figure 1B**). The ASPR was concentrated between 25-84 years of age in both sexes (**Figure 1C**). Three age groups of female patients with higher ASIR, were 5.6/100000 for those aged 40-44 years, 6.2/100000 for those aged 60-64 years, and 5.8/100000 for those aged 95 years, respectively. A more pronounced bimodal distribution was observed in male patients, aged 55-59 years and 85-89 years, respectively (**Figure 1D**).

### Patterns of thyroid cancer burden from 1990 to 2019

**Figure 2:**
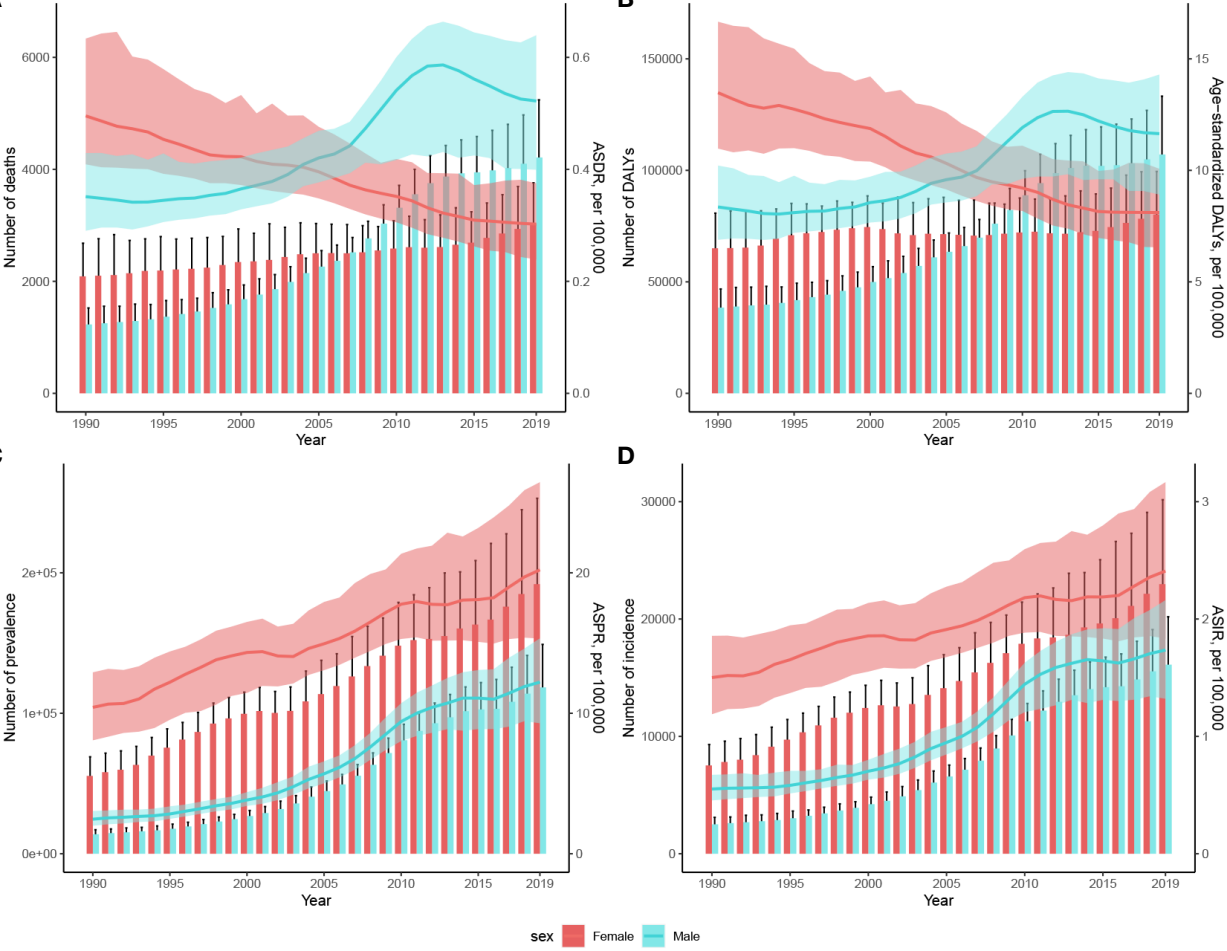
Changes in the number of cases and rates of age-standardized **(A)** mortality, **(B)** DALYs, **(C)** prevalence, and **(D)** incidence of TC in China between 1990 and 2019. Cases are shown using bars while rates are represented with lines for these metrics. The estimated values are displayed with solid lines and the upper and lower boundaries of the 95% UIs are indicated with shading. ASIR, Age-standardized incidence rate; ASDR, Age-standardized deaths rate; ASPR, Age-standardized prevalence rate; DALYs, Disability-adjusted life-years; UIs, Uncertainty intervals.

**Figure 2** demonstrates the trends in thyroid cancer-related mortality, DALYs, prevalence, and ASDRs between the sexes from 1990 to 2019. ASDR and age-standardized DALYs for males and females show the same trend. Namely, ASDR and age-standardized DALYs for females showed a significant downward trend, and ASDR and age-standardized DALYs for males showed an upward trend in 2013, and then declined annually. The number of TC-related deaths in female patients showed a small increase, and the number of deaths in male patients increased significantly, exceeding the number of females after 2006. There was no significant trend in the number of DALYs due to TC in females, whereas male patients showed an increasing trend and exceeded female patients after 2007 (**Figures 2A, B**). In both male and female, the prevalence and incidence of TC showed an increasing trend between 1990 and 2019 in terms of number, ASPR, and ASIR (**Figures 2C, D**).

### Thyroid cancer deaths and DALYs by high body-mass index

**Figure 3:**
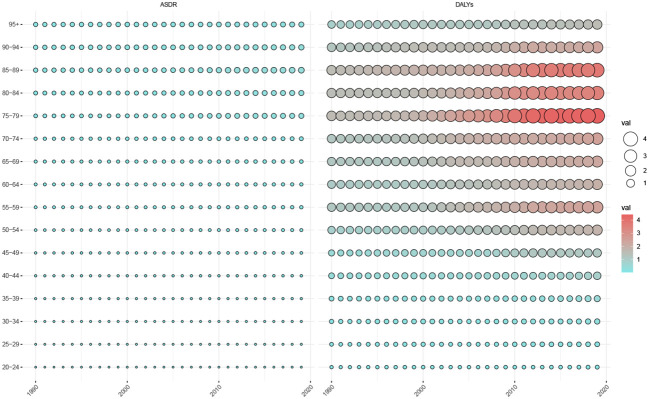
Trends in ASDR and age-standardized DALYs in patients with thyroid cancer attributed to HBMI by age in the total population, 1990-2019. ASDR, Age-standardized deaths rate; DALYs, Disability-adjusted life-years.

**Figure 3** shows the changes in the distribution of overall TC ASDRs and DALYs attributed to HBMI across age groups between 1990 and 2019. Between 1990 and 2019, there was a slower upward trend in TC ASDRs attributed to HBMI in patients older than 70 years, with a smaller trend observed in patients younger than 70 years. However, during the last decade, TC DALYs attributed to HBMI have risen to varying degrees in all age groups, particularly in the 75-79-years and 85-89-years age groups.

**Figure 4:**
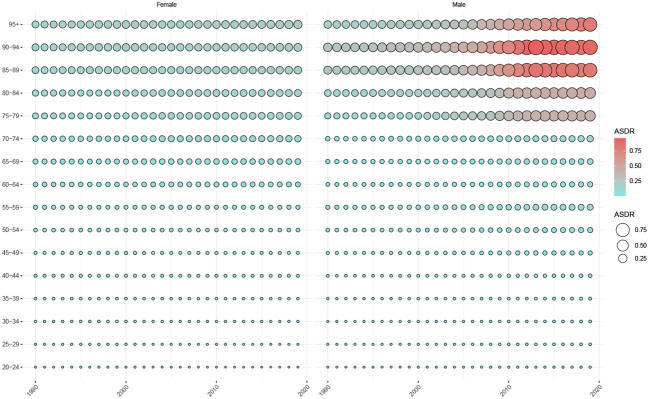
Trends in ASDR in patients with thyroid cancer attributed to HBMI, by sex and age group, 1990-2019. ASDR, Age-standardized deaths rate.

**Figure 4** shows the trend of ASDR in patients with TC attributed to HBMI and grouped by sex and age. From 1990 to 2019, the ASDR for HBMI thyroid cancer in women slowly increased in patients aged > 55 years. In contrast, among male patients, the ASDR for HBMI thyroid cancer in patients aged > 74 years increased rapidly in the past decade.

**Figure 5:**
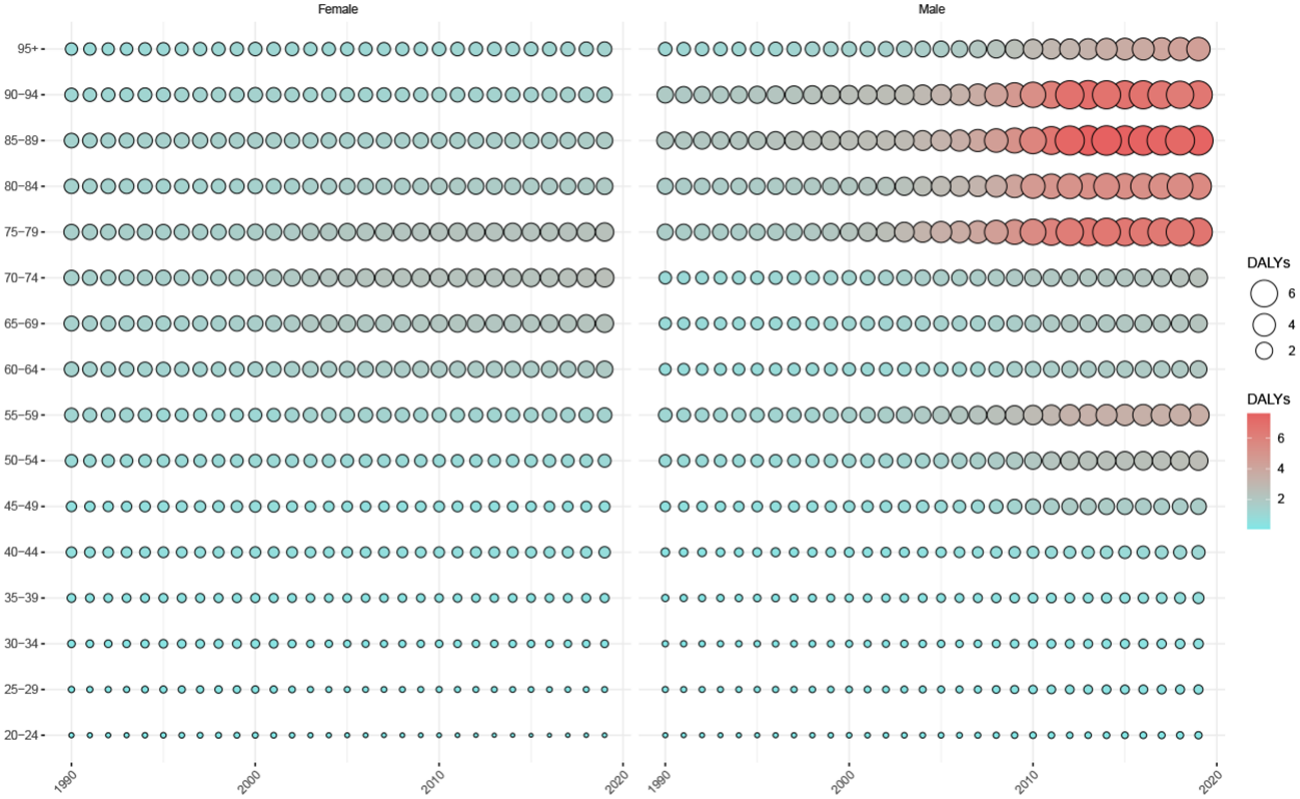
Trends in age-standardized DALYs in patients with thyroid cancer attributed to HBMI, by sex and age group, 1990-2019. DALYs, Disability-adjusted life-years.

**Figure 5** shows the trend in DALYs in patients with TC attributed to HBMI between 1990 and 2019. As observed from the size and density of the balloon plot, female patients with TC aged 60-80 years showed an increasing trend in DALYs for TC associated with HBMI over the last decade, with little change in other age groups. Male patients showed an increasing trend in all age groups, particularly in those aged 50-59 years and >75 years.

The bubble chart shows that age-standardized YLDs for TC attributed to HBMI showed no significant change across all ages between 1990 and 2019. In recent years, the age-standardized YLLs for TC attributed to HBMI showed an increasing trend in TC disease burden in China among patients over 50 years of age, particularly among patients aged 75-79 years (**Supplementary Figure 1A**). Age-standardized YLLs for TC attributed to HBMI in women have changed little overall but showed an increasing trend over time in women aged 60 to 80 years of age. Male patients younger than 45 years showed little change in YLLs in recent years and showed an increasing trend between 45-59 and > 75 years; the rate of YLL increase was greater above the age of 75 years (**Supplementary Figure 1B**). In terms of age-standardized YLDs for TC attributable to HBMI, YLDs have exhibited increase among both sexes, particularly among female patients aged 35-79 years and male patients aged > 30 years (**Supplementary Figure 1C**).

## Discussion

This study describes the long-term trends in the ASDR, ASIR, ASPR, and age-standardized DALYs for TC in China between 1990 and 2019. Over the past 30 years, significant upward trends have been observed in ASIR and ASPR, with a declining trend in age-standardized DALYs, and non-significant changes in ASDR. The rapid increase in the incidence of TC may be attributed to the application of new imaging techniques for thyroid evaluation (27). This does not exclude the possibility of an actual increase in TC incidence. However, one study noted that the ASIR for patients with TC in the United States did not change significantly between 2011-2019, while the burden of TC appeared to be declining in over 15 countries in the EU (28). Studies have also indicated that TC incidence, mortality, DALYs, and ASIR have shown varying degrees of increase worldwide, with decreasing trends in the ADSR and age-standardized DALYs rates (3). The difference in results across studies may be due to sociodemographic index (SDI) levels, with high SDI regions exhibiting a progressive increase in ASIR until 2010 followed by a decrease (3). Age-standardized DALYs in Chinese patients with TC showed a decreasing trend, consistent with other studies (3, 29). However, unlike other studies, no significant changes in the ASDR were observed in this study.

This study found that there was a noteworthy increase in the TC burden according to prevalence, incidence, mortality, and DALYs among male patients. There are several possible reasons for this discrepancy. First, women have a higher prevalence of benign thyroid disease and are more likely to undergo thyroid screening and early diagnosis. Furthermore, over the past decades, TC cases have occurred mainly among menopausal women, and the age of menopause in women is considered an independent factor in papillary TC (30). Nilubol et al. concluded that male patients with TC were older and more likely to have advanced and aggressive disease (31). Lower specific survival rates have been reported in men but not women with TC in areas of lower socioeconomic status (32). Men are more likely to have advanced TC at the time of diagnosis leading to early specific death, which may be explained by differences between the sexes in biology and behavioral attitudes towards medical care (33–35). While female TC incidence rates have been higher than those in men since 2004, male mortality rates exceed those in women and continue to rise, suggesting the need to enhance early detection and timely treatment of TC in men. In addition, the age-standardized YLD rates increased significantly in both men and women, suggesting an increased burden of disease and disability due to TC.

The prevalence and incidence of TC in China have increased over the last 30 years, particularly in the male population. Socioeconomic level plays an important role in determining the incidence and prevalence of TC. Previous studies have shown that the SDI is closely related to the prevalence and incidence of TC, with an increasing trend in TC prevalence in regions and countries with moderate or low SDI (3, 36). GBD data show an upward trend in SDI in China, increasing from 0.456 in 1990 to 0.707 in 2017. Since 2003, China’s SDI has exceeded the global average (37). The increase observed in the annual incidence and number of patients over the 30-year period of this study may be explained by advances in TC diagnosis as well as the increase in the total population in China. Therefore, a greater focus is required on TC preventive strategies in China in parallel with rapid socioeconomic improvements. We also observed a decreasing trend in mortality and age-standardized DALYs in female patients with TC. Conversely, increasing and decreasing trends were observed in male patients with rates surpassing those observed in female patients around 2006. The introduction of multidisciplinary and individualized treatments has greatly improved the prognosis of TC over the last decade, and the upward and downward trends may be an effect of the implementation of TC treatment guidelines (38). Considering the high life expectancy of individuals with TC as well as their active contribution to society and their important role in caring for their families, it is important to develop targeted programs to effectively reduce the burden of TC.

According to this study, in 2019, the ASDR and age-standardized DALYs were higher in male patients with TC than those in female patients. It is recognized that specific biological characteristics are associated with TC in women while in men, obesity-related TC occurs more frequently (39, 40). This could explain the recent increase in the incidence and mortality indicators of TC in men. Age is also a key factor that influences the occurrence, progression, and prognosis of TC. Overall, morbidity and mortality rates increase significantly with age, and the elderly population, particularly men aged ≥80 years, has the highest burden of TC. Considering that globally China has the largest aging population, TC prevention and treatment in elderly men should receive increased attention. The delivery of health education, nutritional support, surgical treatment, intensive care, and comorbidity management in elderly patients with TC should be improved.

TC resulting from obesity occurs in countries or regions with high social development, European countries like Italy (41) and the United States (42) and HBMI as a significant risk factor for TC was not earlier emphasized in China. This study confirmed that increased BMI was associated with a greater risk of TC in both men and women, with a stronger correlation in men (43, 44). Our study found that high BMI has played an increasingly important role in the TC burden of disease in China in recent years. Studies have shown that high BMI is associated with a higher risk of BRAF mutant TC (45). While there is a dose-response relationship between obesity and TC, the prevalence of obesity is closely related to the level of socioeconomic development. As China continues to modernize and urbanize, the prevalence of obesity and its associated health burdens are increasing (46). Therefore, as a potential modifiable risk factor, health education and public health strategies for controlling high BMI may be beneficial for reducing the disease burden of TC.

This study has several limitations. First, because the GBD compiles data at the national or regional level, we were unable to analyze the distribution of the TC disease burden between provinces and urban-rural areas. Further data are required to provide detailed information on the burden of TC in China. Second, although inadequate or unreliable diagnoses of TC were corrected using redistribution algorithms, the accuracy of the diagnosis remains limited. Additionally, information biases regarding the epidemiological evaluation of TC are inevitable, such as the scarcity of data on ethnic minorities. Finally, the GBD did not conduct further investigations into the histological characteristics, grade, and risk factors of TC stratified by subgroups.

## Conclusions

In summary, this study presents the current disease burden of TC and its long-term trends in China over the past 30 years using the ASDR, age-standardized DALYs, ASPR, and ASIR indicators. The disease burden of TC in China increased between 1990 and 2019, particularly among elderly men. Additionally, in recent years, HBMI has played an increasing role in mortality and DALYs associated with TC in men. Further research and targeted measures are needed to optimize the prevention, diagnosis, treatment and management of TC to reduce the disease burden.

## Author’s note

Contributions to the manuscript were made by all authors, and permission was granted for the submitted version.

## Data availability statement

The datasets presented in this study can be found in online repositories. The names of the repository/repositories and accession number(s) can be found below: GBD data are available for free download for researchers, and anyone can filter and download the data using the official visualization website (https://vizhub.healthdata.org/gbd-results/).

## Author contributions

SJ and KX: study design. SJ and KX: data collection, data analysis, and revision. LL and XX: First draft writing and revision. SJ and LL: language editing. All authors contributed to the article and approved the submitted version.

## Supplementary material

Supplementary Figure 1
**(A)** Trends in age-standardized YLDs and YLLs for TCs attributed to high body-mass index between 1990 and 2019. YLDs: Years lived with disability; YLL: Years of life lost; TC: Thyroid cancer. **(B)** Trends in age-standardized YLLs for TC attributable to high body mass index by gender, 1990 to 2019. YLDs: Years lived with disability; TC: Thyroid cancer. **(C)** Trends in age-standardized YLDs for TC attributable to high body mass index by gender, 1990 to 2019. YLL, Years of life lost; TC, Thyroid cancer.
