# Supplementary figures and images for: Thyroid cancer burden and risk factors in China from 1990-2019: a systematic analysis using the global burden of disease study
Source: Front Oncol. 2023 Nov 8;13:1231636. doi: 10.3389/fonc.2023.1231636 (PMC10663347; doi:10.3389/fonc.2023.1231636)

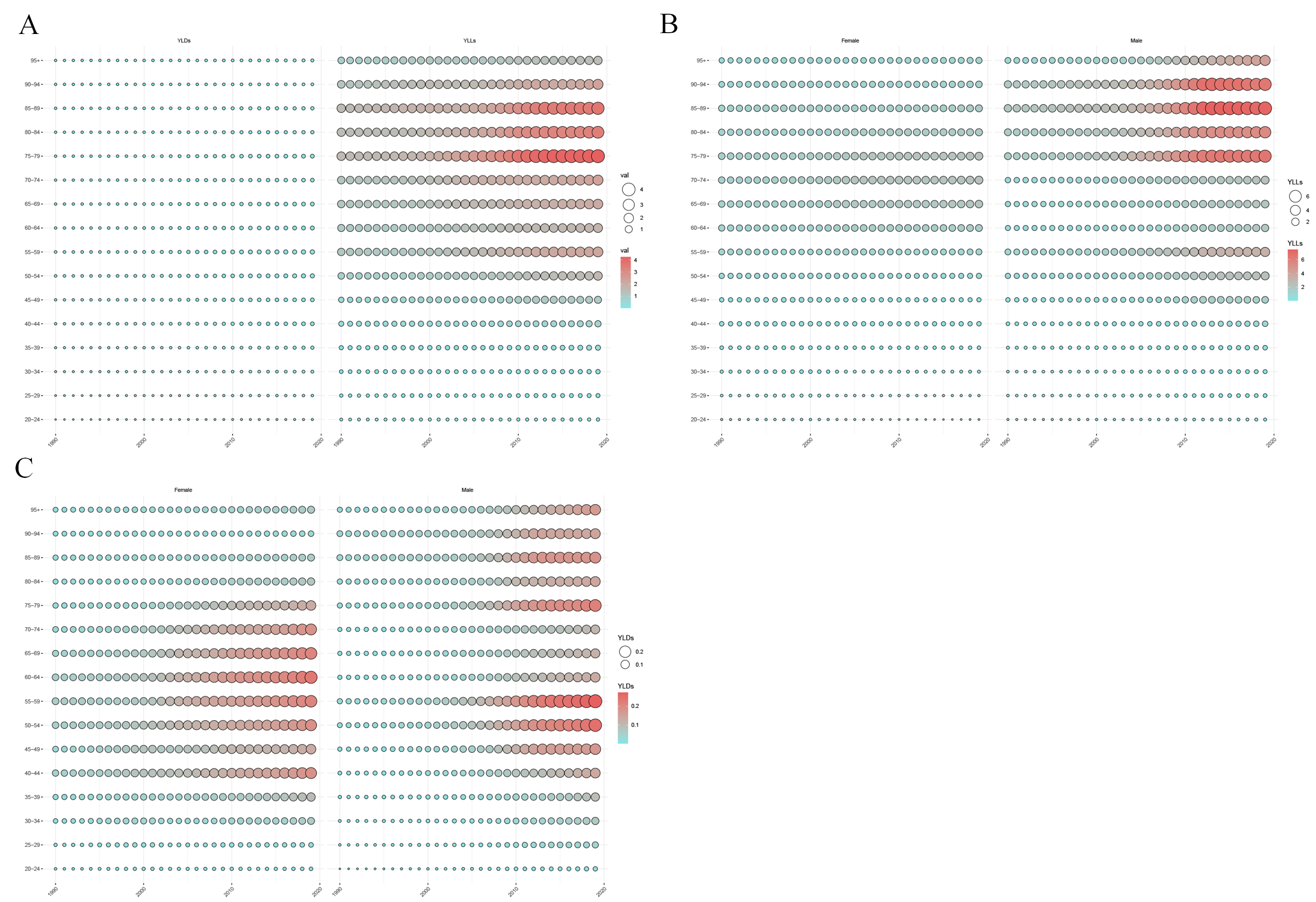

Supplement: Supplementary Figure 1 — (A) Trends in age-standardized YLDs and YLLs for TCs attributed to high body-mass index between 1990 and 2019. YLDs: Years lived with disability; YLL: Years of life lost; TC: Thyroid cancer. (B) Trends in age-standardized YLLs for TC attributable to high body mass index by gender, 1990 to 2019. YLDs: Years lived with disability; TC: Thyroid cancer. (C) Trends in age-standardized YLDs for TC attributable to high body mass index by gender, 1990 to 2019. YLL, Years of life lost; TC, Thyroid cancer. [file Image_1.tif]
